# Supplementary material for: Gene Expression and Drug Sensitivity Analysis of Mitochondrial Chaperones Reveals That HSPD1 and TRAP1 Expression Correlates with Sensitivity to Inhibitors of DNA Replication and Mitosis
Source: Biology (Basel). 2023 Jul 11;12(7):988. doi: 10.3390/biology12070988 (PMC10376793; doi:10.3390/biology12070988)

Supplementary Table S1. Mitochondrial chaperone genes used for the paper

GeneID	ENSID	Symbol	Gene Name
10845	ENSG00000166855	CLPX	caseinolytic mitochondrial matrix peptidase chaperone subunit(CLPX)
3313	ENSG00000113013	HSPA9	heat shock protein family A (Hsp70) member 9(HSPA9)
10939	ENSG00000141385	AFG3L2	AFG3 like matrix AAA peptidase subunit 2(AFG3L2)
9093	ENSG00000103423	DNAJA3	DnaJ heat shock protein family (Hsp40) member A3(DNAJA3)
9361	ENSG00000196365	LONP1	lon peptidase 1, mitochondrial(LONP1)
150274	ENSG00000100209	HSCB	HscB mitochondrial iron-sulfur cluster cochaperone(HSCB)
3336	ENSG00000115541	HSPE1	heat shock protein family E (Hsp10) member 1(HSPE1)
27429	ENSG00000115317	HTRA2	HtrA serine peptidase 2(HTRA2)
3329	ENSG00000144381	HSPD1	heat shock protein family D (Hsp60) member 1(HSPD1)
10730	ENSG00000136758	YME1L1	YME1 like 1 ATPase(YME1L1)
6687	ENSG00000197912	SPG7	SPG7, paraplegin matrix AAA peptidase subunit(SPG7)
8192	ENSG00000125656	CLPP	caseinolytic mitochondrial matrix peptidase proteolytic subunit(CLPP)
134266	ENSG00000164284	GRPEL2	GrpE like 2, mitochondrial(GRPEL2)
131118	ENSG00000205981	DNAJC19	DnaJ heat shock protein family (Hsp40) member C19(DNAJC19)
10131	ENSG00000126602	TRAP1	TNF receptor associated protein 1(TRAP1)

Supplementary Figure S1. **A)** Z-Score distribution. Vertical dotted lines denote the cutoff (Z-score beyond $\pm$ 1.7). **B)** Distribution of the Res-Score$_{global}$. The black dotted line indicates the chosen significance cutoff of 7.9. **C)** Distribution of the Sen-Score$_{global}$. The black dotted line indicates the chosen significance cutoff of 8.42.

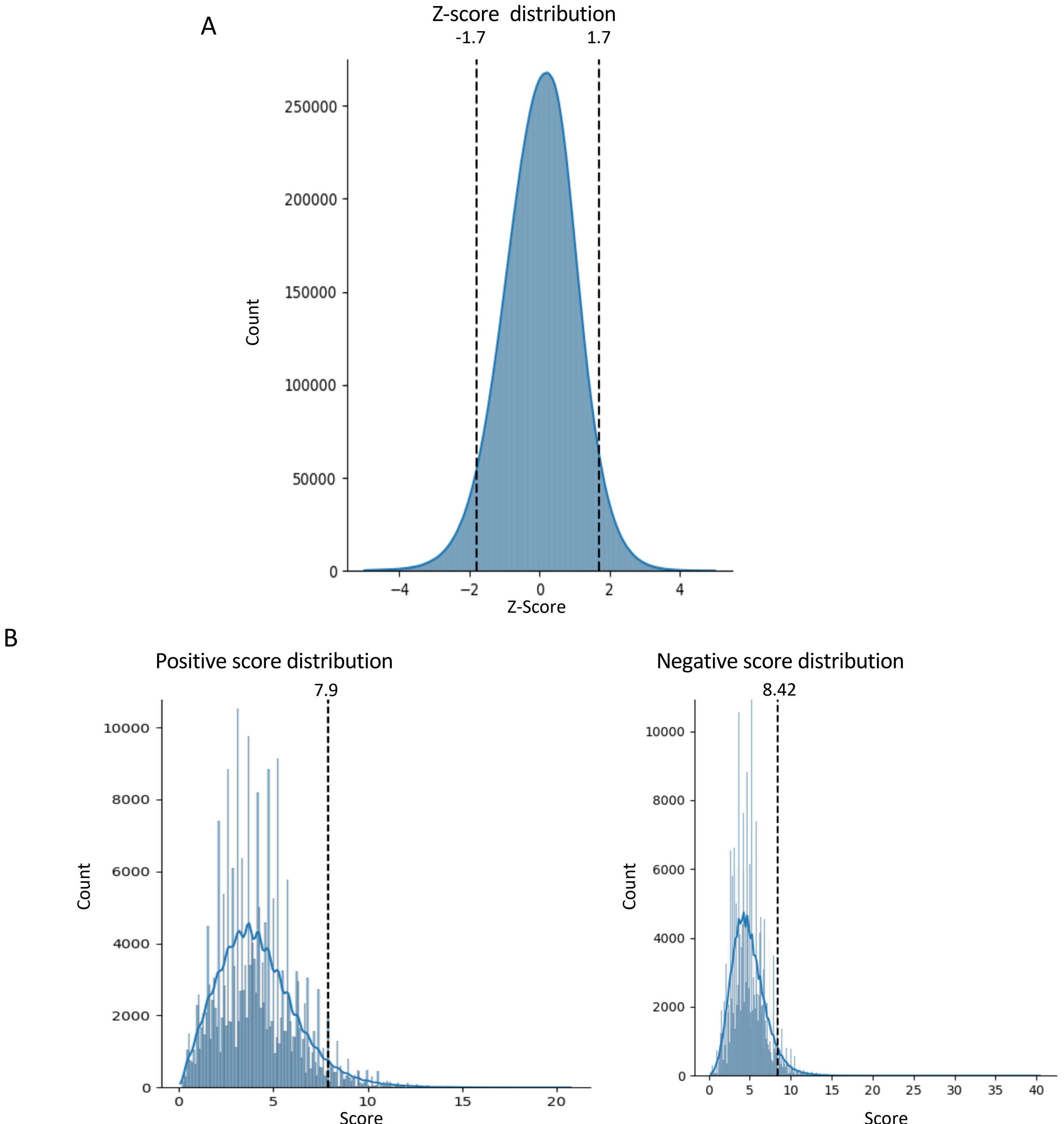

Supplementary Figure S2. A) Bi-directional bar chart showing the $Score_{global}$ of CLPP in all drug categories. The blue bar denotes Sen-$Score_{global}$; the red bar denotes Res-$Score_{global}$. The black dotted line indicates the cutoff for significant scores. **B)** Bubble plot showing the percentage of drugs with significant negative Zscore in each drug category across all cancer subtypes. **C)** Bubble plot showing the percentage of drugs with significant positive Zscore in each drug category across all cancer subtypes.

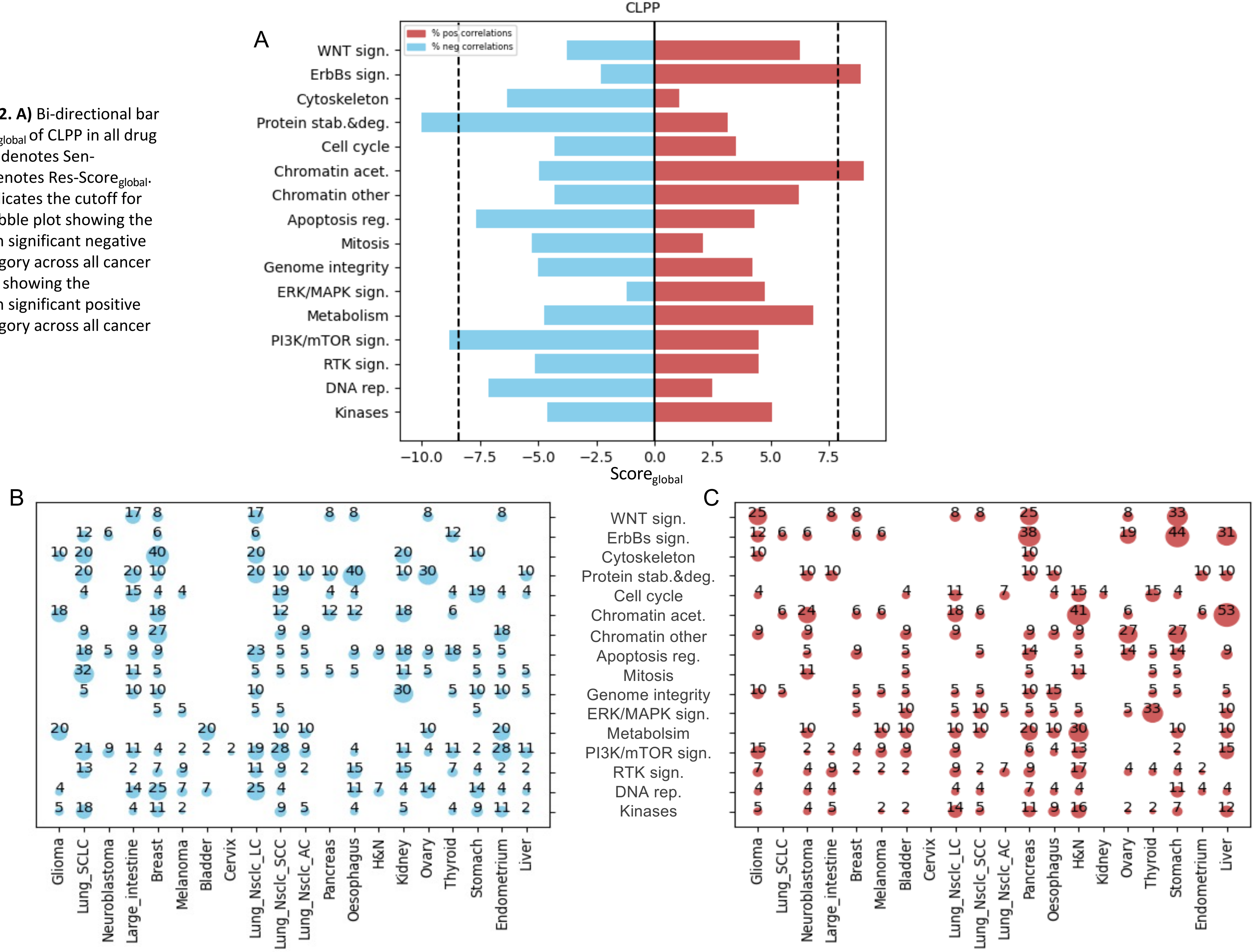

Supplementary Figure S3. A) Bi-directional bar chart showing the $Score_{global}$ of SPG7 in all drug categories. The blue bar denotes Sen-$Score_{global}$; the red bar denotes Res-$Score_{global}$. The black dotted line indicates the cutoff for significant scores. **B)** Bubble plot showing the percentage of drugs with significant negative Zscore in each drug category across all cancer subtypes. **C)** Bubble plot showing the percentage of drugs with significant positive Zscore in each drug category across all cancer subtypes.

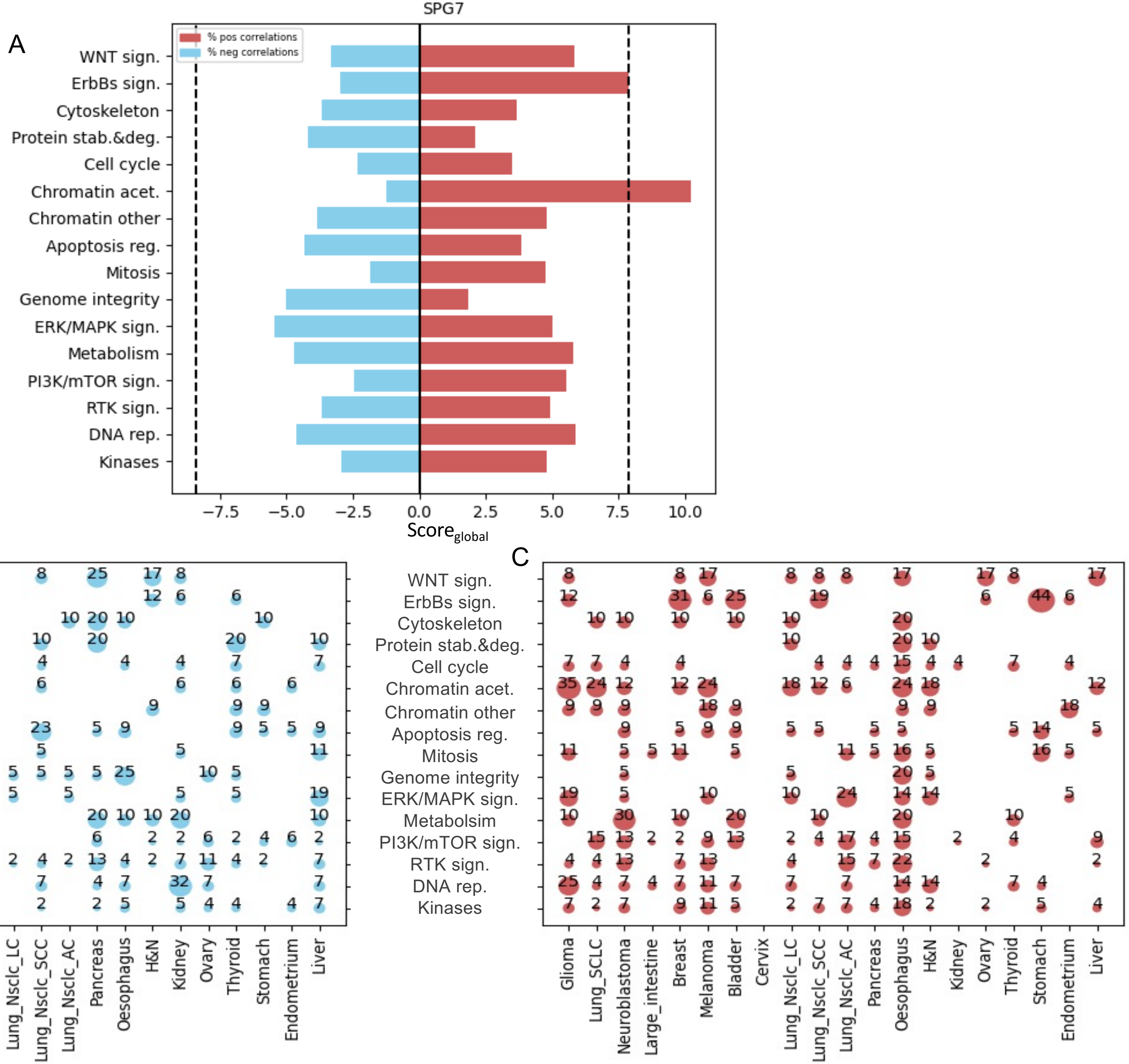

Supplementary Figure S4. A) Bi-directional bar chart showing the $Score_{global}$ of HTRA2 in all drug categories. The blue bar denotes Sen-$Score_{global}$; the red bar denotes Res-$Score_{global}$. The black dotted line indicates the cutoff for significant scores. **B)** Bubble plot showing the percentage of drugs with significant negative Zscore in each drug category across all cancer subtypes. **C)** Bubble plot showing the percentage of drugs with significant positive Zscore in each drug category across all cancer subtypes.

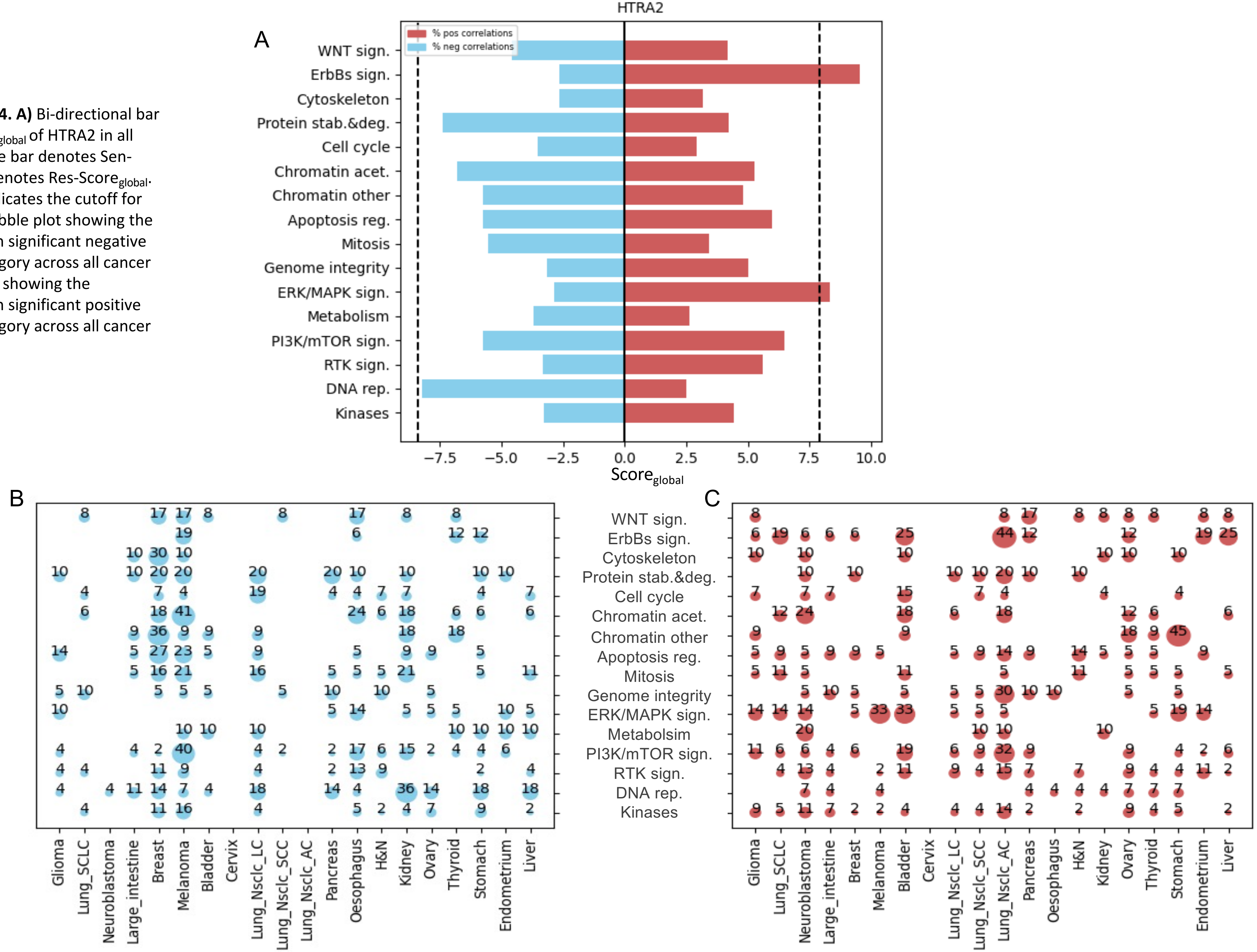

Supplementary Figure S5. A) Bi-directional bar chart showing the $Score_{global}$ of LONP1 in all drug categories. The blue bar denotes Sen-$Score_{global}$; the red bar denotes Res-$Score_{global}$. The black dotted line indicates the cutoff for significant scores. **B)** Bubble plot showing the percentage of drugs with significant negative Zscore in each drug category across all cancer subtypes. **C)** Bubble plot showing the percentage of drugs with significant positive Zscore in each drug category across all cancer subtypes.

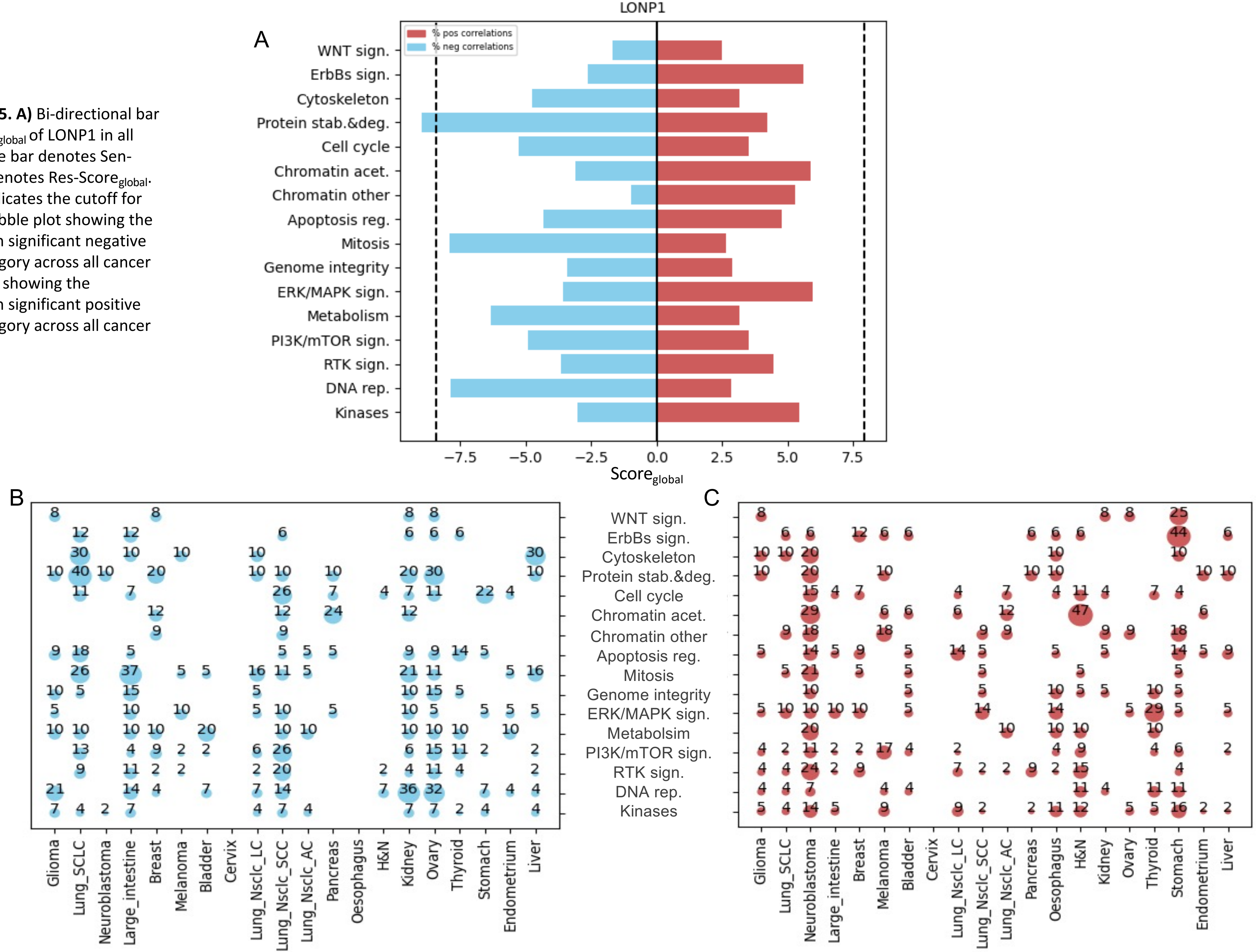

Supplementary Figure S6. A) Bi-directional bar chart showing the $Score_{global}$ of HSPA9 in all drug categories. The blue bar denotes Sen-$Score_{global}$; the red bar denotes Res-$Score_{global}$. The black dotted line indicates the cutoff for significant scores. **B)** Bubble plot showing the percentage of drugs with significant negative Zscore in each drug category across all cancer subtypes. **C)** Bubble plot showing the percentage of drugs with significant positive Zscore in each drug category across all cancer subtypes.

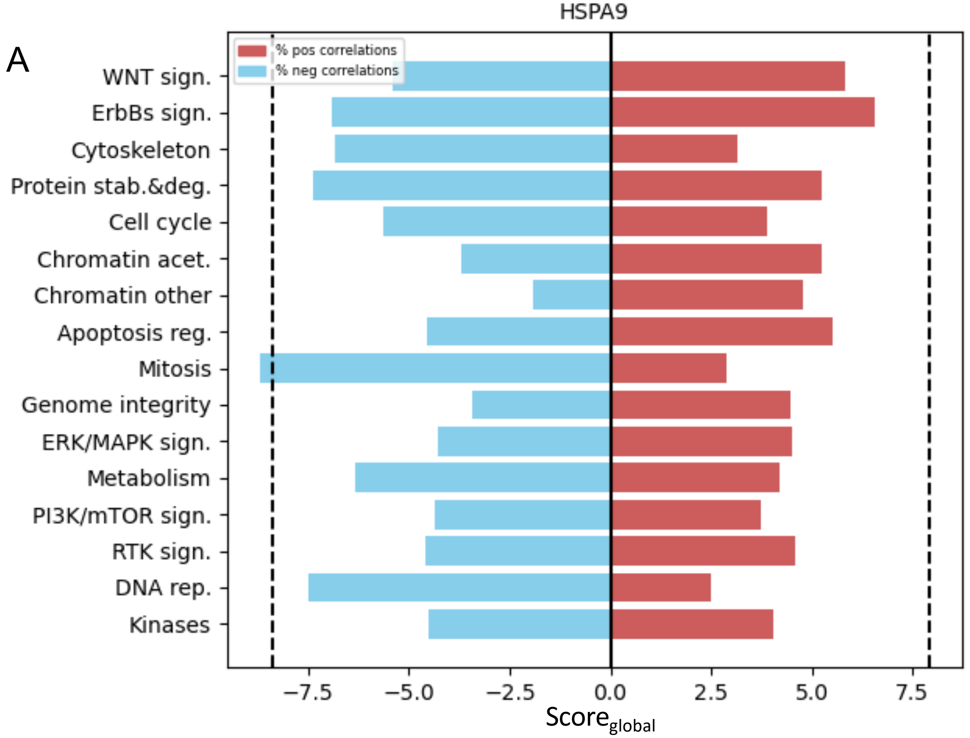

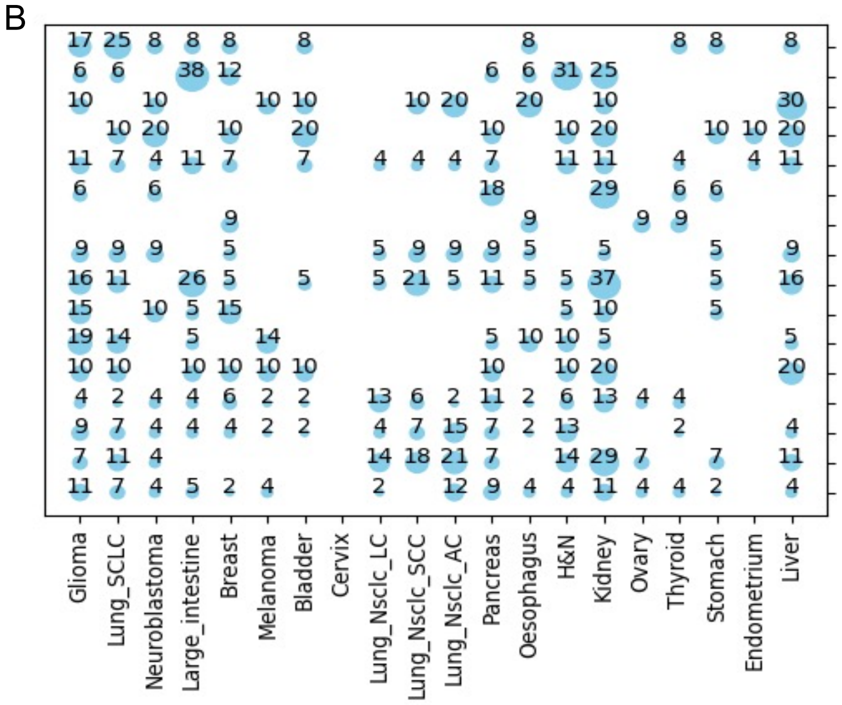

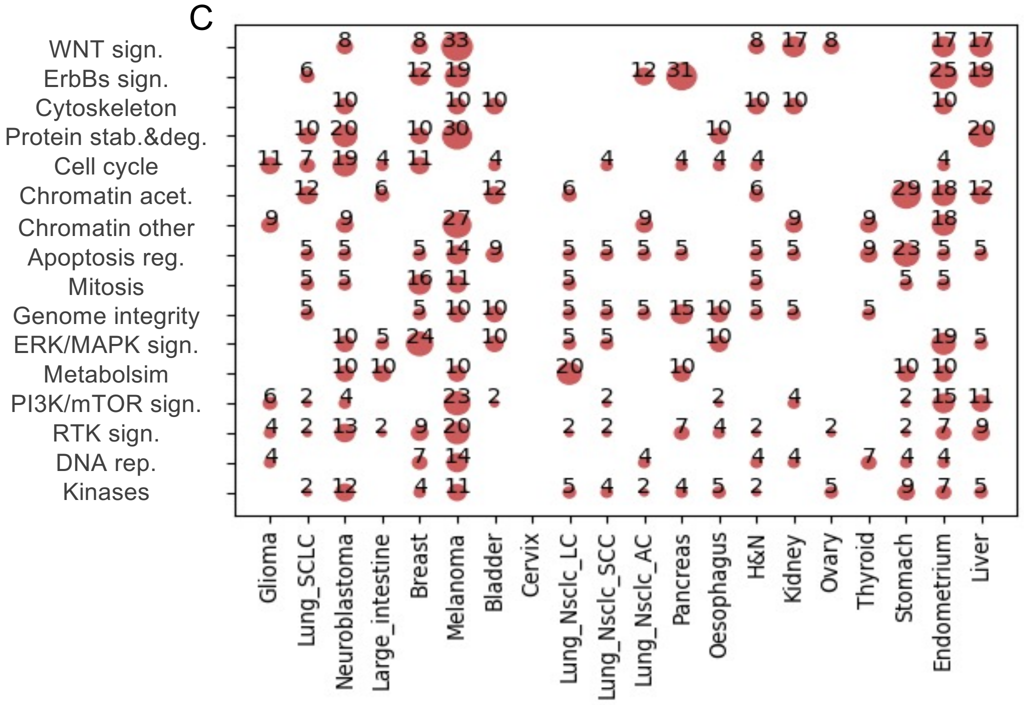

Supplementary Figure S7. A) Bi-directional bar chart showing the $Score_{global}$ of HSPE1 in all drug categories. The blue bar denotes Sen-$Score_{global}$; the red bar denotes Res-$Score_{global}$. The black dotted line indicates the cutoff for significant scores. **B)** Bubble plot showing the percentage of drugs with significant negative Zscore in each drug category across all cancer subtypes. **C)** Bubble plot showing the percentage of drugs with significant positive Zscore in each drug category across all cancer subtypes.

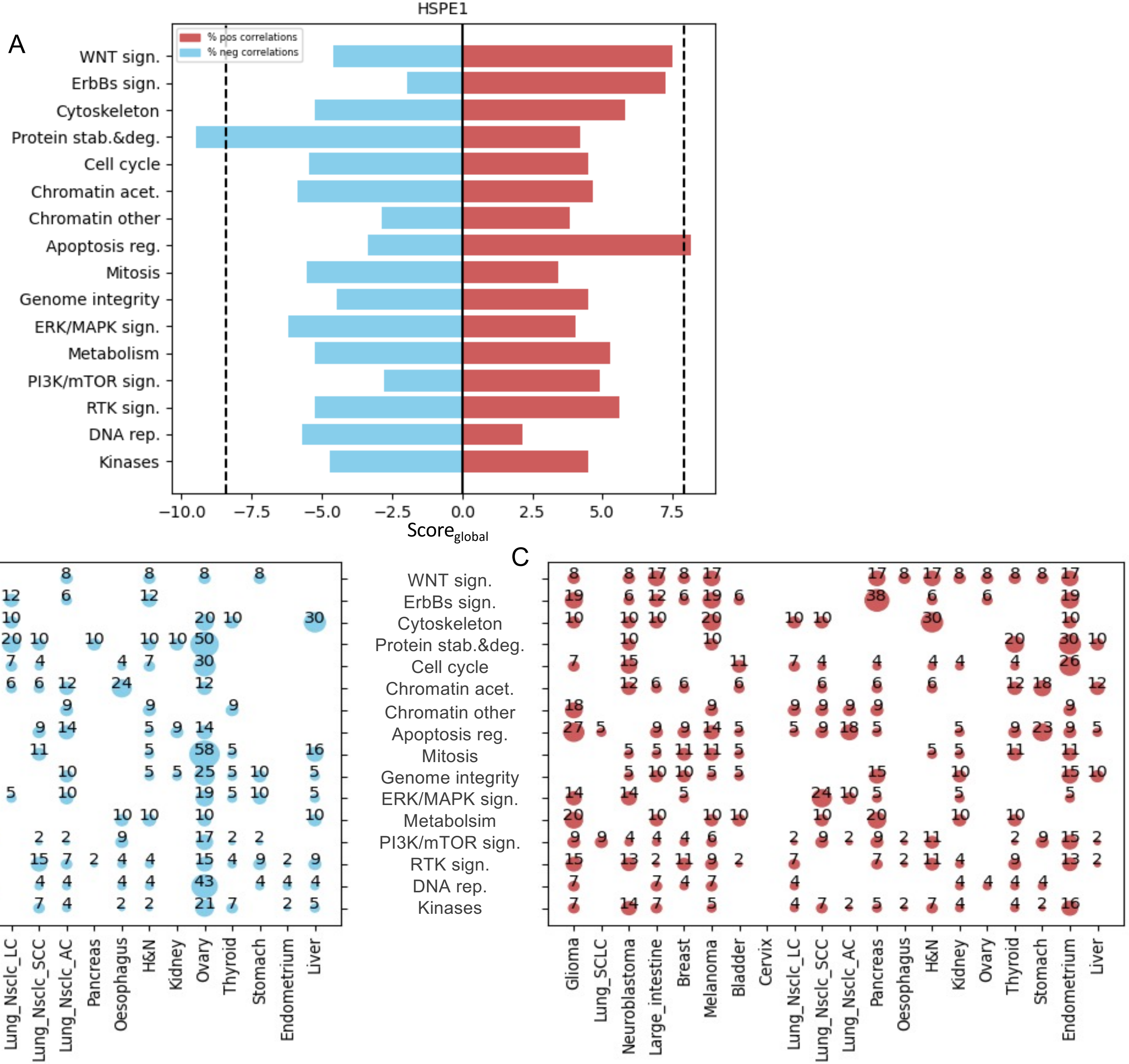

Supplementary Figure S8. A) Bi-directional bar chart showing the $Score_{global}$ of AFG3L2 in all drug categories. The blue bar denotes Sen-$Score_{global}$; the red bar denotes Res-$Score_{global}$. The black dotted line indicates the cutoff for significant scores. **B)** Bubble plot showing the percentage of drugs with significant negative Zscore in each drug category across all cancer subtypes. **C)** Bubble plot showing the percentage of drugs with significant positive Zscore in each drug category across all cancer subtypes.

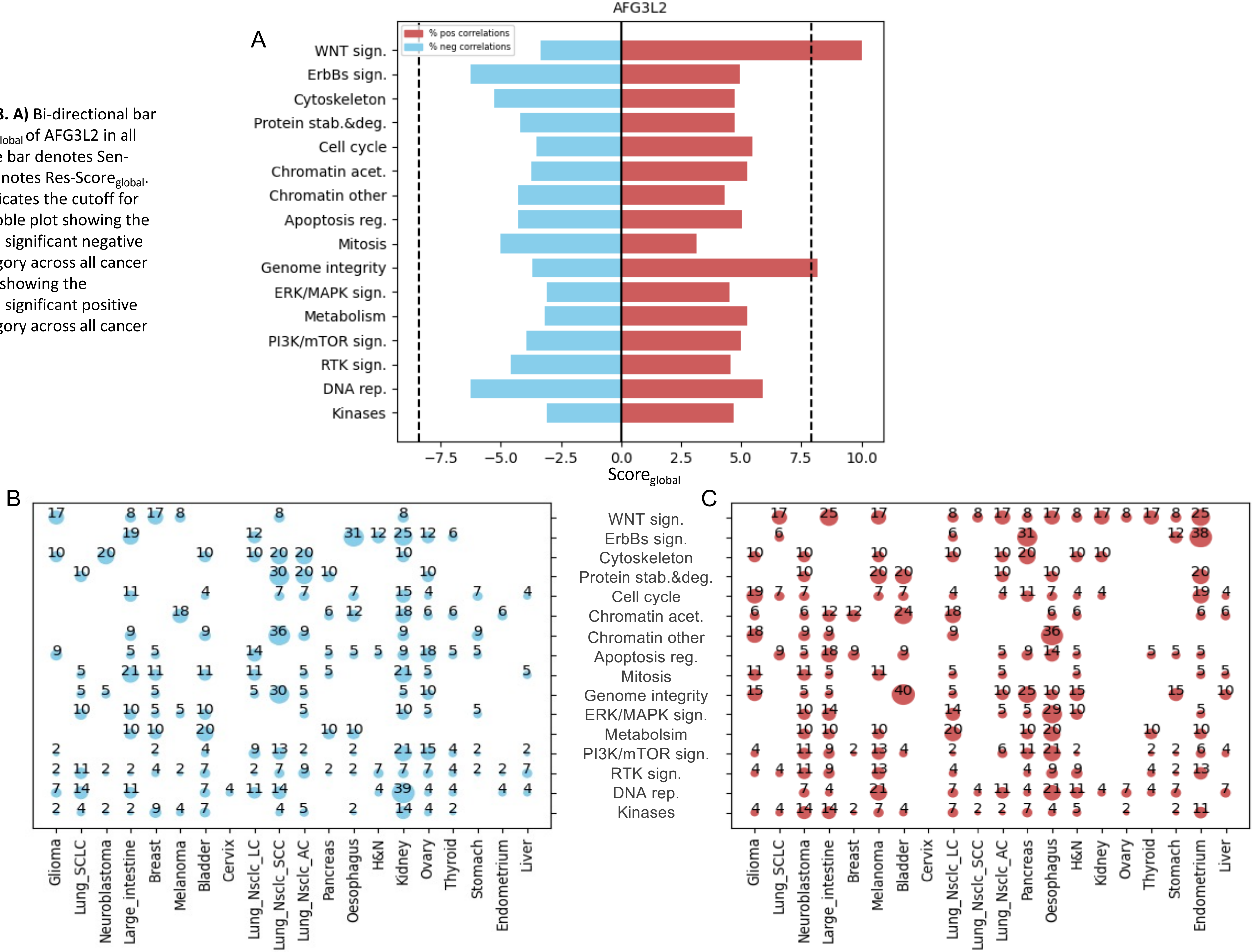

Supplementary Figure S9. Original WB images.

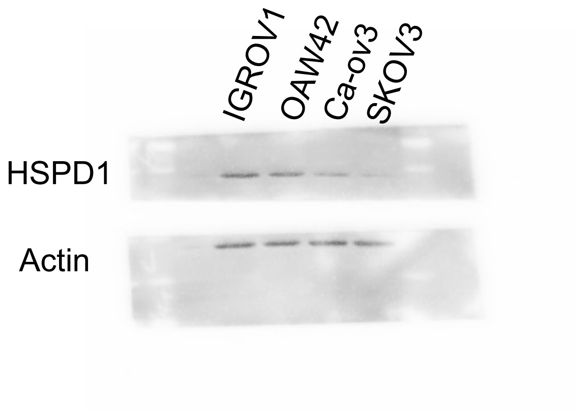

Supplement: Supplementary file 1 [file biology-12-00988-s001.zip › biology-2491761-supplementary (1).pdf]
